# RXFP1 is Targeted by Complement C1q Tumor Necrosis Factor-Related Factor 8 in Brain Cancer

**DOI:** 10.3389/fendo.2015.00127

**Published:** 2015-08-13

**Authors:** Thatchawan Thanasupawat, Aleksandra Glogowska, Maxwell Burg, G. William Wong, Cuong Hoang-Vu, Sabine Hombach-Klonisch, Thomas Klonisch

**Affiliations:** ^1^Department of Human Anatomy and Cell Science, Faculty of Health Sciences, College of Medicine, University of Manitoba, Winnipeg, MB, Canada; ^2^Department of Physiology, Center for Metabolism and Obesity Research, Johns Hopkins University School of Medicine, Baltimore, MD, USA; ^3^Clinics of General, Visceral and Vascular Surgery, Martin Luther University, Halle/Salle, Germany; ^4^Department of Obstetrics, Gynecology and Reproductive Medicine, Faculty of Health Sciences, College of Medicine, University of Manitoba, Winnipeg, MB, Canada; ^5^Department of Surgery, Faculty of Health Sciences, College of Medicine, University of Manitoba, Winnipeg, MB, Canada; ^6^Department of Medical Microbiology and Infectious Diseases, Faculty of Health Sciences, College of Medicine, University of Manitoba, Winnipeg, MB, Canada

**Keywords:** C1q/TNF-related proteins, RXFP1, CTRP8, cancer, brain tumor

## Abstract

The relaxin-like RXFP1 ligand–receptor system has important functions in tumor growth and tissue invasion. Recently, we have identified the secreted protein, CTRP8, a member of the C1q/tumor necrosis factor-related protein (CTRP) family, as a novel ligand of the relaxin receptor, RXFP1, with functions in brain cancer. Here, we review the role of CTRP members in cancers cells with particular emphasis on CTRP8 in glioblastoma.

## Relaxin-Like Peptides and Cognate Receptors in Cancer: A Brief Overview

Most of the cellular and molecular mechanisms involved in RXFP1-mediated cancer promotion have been established in breast, thyroid, and prostate cancer models. For more detailed reviews on relaxin-like peptides and their cognate receptors, the reader is referred to recent excellent reviews (1–5). Increased expression of relaxin-like peptides has been detected in *breast cancer* (6, 7). Using the ERα-positive human breast cancer cell line MCF-7, the group of Mario Bigazzi showed that highly purified porcine relaxin acted in a dose- and time-dependent manner and promoted proliferation only with short-term exposure at low concentrations. Long-term exposure over up to 7 days reduced proliferation and promoted differentiation of MCF-7 cells as demonstrated with up-regulation of cell surface protein E-cadherin (8, 9). This was accompanied by an increase in inducible NO synthase activity and intracellular NO production (10). In co-culture with myoepithelial cells, relaxin enhanced ultrastructural signs of MCF-7 cell differentiation (11). Exposure to human recombinant RLN2 for 24 h induced S100A4 expression and increased cell migration in ERα-negative MDA-MB-231 triple-negative breast cancer cells, but exposure for more than 3 days downregulated S100A4 levels and reduced cell migration and invasiveness in the same cell model in an RXFP1-dependent manner, leading to reduced tumor xenograft growth *in vivo* (12). In an *in vitro* brain metastasis model, RLN2 promoted the invasion of RXFP1-expressing MCF-7 human breast cancer cells into brain tissue slices (13). These data suggest concentration-, time-, and cell context-dependent actions of relaxin in breast cancer and an essential role for RXFP1 in mediating cell motility and invasion.

Increased expression of RLN2 and RXFP1 was also shown in *thyroid cancer*. RLN2/RXFP1 signaling promotes thyroid cancer motility and invasiveness. RXFP1 mediated the motility-enhancing effect of RLN2 via induction of S100A4 in human thyroid carcinoma cells and RLN2 enhanced thyroid xenograft angiogenesis (14). RLN2/RXFP1 signaling increased the expression and secretion of the lysosomal proteinases, cathepsin-D and cathepsin-L, resulting in enhanced elastinolytic activity and cell invasion through elastin matrices (15). RXFP1 activation by RLN2 in human thyroid cancer cells increased cell migration and extracellular matrix invasion resulting from enhanced collagenolytic activity through the upregulation of MMP2 and MT1-MMP/MMP14 and the increased secretion of MMP2 (16).

In *prostate cancer*, RLN2/RXFP1 signaling increased cell migration and proliferation in androgen-receptor (AR)-dependent LNCaP and AR-independent PC3 prostate cancer cells (17) and promoted growth in xenografts derived of androgen-independent PC3 prostate cancer cells (18). The siRNA-mediated knockdown of RXFP1 prevented the RLN2-induced increase in prostate cancer cell proliferation and invasiveness and induced apoptosis (19). Injection of siRNA-loaded biodegradable nanoparticles into xenografts of AR-positive LNCaP cells and AR-negative PC3 cells downregulated RXFP1 and resulted in a significant reduction in tumor proliferation and metastasis, implicating RXFP1 as an important growth and survival factor in prostate cancer (20). RXFP1-dependent and RLN2-induced proliferation of prostate carcinoma cells was mediated via a PI3K/Akt signaling pathway. Simultaneous blocking of protein kinase A (PKA) and NF-κB signaling almost completely abolished RLN2-mediated proliferation and colony formation in LNCaP cells (21). The extracellular N-terminal low density lipoprotein A (LDL-A) module of RXFP1 was shown to reduce S100A4, S100P, IGFBP2, and MUC1 expression and inhibit RXFP1-mediated proliferation and invasion of PC3 prostate cancer cells. Similar to RXFP1 knockdown in PC3 cells, LDL-A expression reduced pAKT^T308^ and decreased cell proliferation and colony formation, suggesting LDL-A to block activation of endogenous RXFP1 in PC3 cells (22).

The established role of RXFP1 in cancer and other diseases has prompted attempts to identify specific agonists and antagonists of RXFP1. A recent large high-throughput screen of small molecule libraries yielded RXFP1 agonists (23, 24). The challenging search for RXFP1 antagonists has so far produced few promising candidates. The conversion of the two arginine residues (B13, B17) to lysines (ΔH2) within the receptor binding domain of the B-chain of human RLN2 (“GRELVR”) was shown to reduce bioactivity and cAMP production in RXFP1-positive myelo-monocytic THP1 cells and RXFP1 expressing HEK293 cells. This ΔH2 mutant was able to bind to RXFP1 and function as a partial antagonist to functional RLN2 in an *in vivo* xenograft model of prostate cancer (25). In MCF-7 cancer cells and renal myofibroblasts endogenously expressing relaxin, the ΔH2 analog blocked RXFP1 activation and significantly inhibited RLN2-induced MCF-7 cell migration (26). When a chemically synthesized ΔH2 antagonist, named AT-001, was used alone or in combination with the anti-mitotic taxane drug docetaxel, xenografts derived from PC3 androgen-independent prostate cancer cells were reported to show a dramatic 60 and 90% reduction in growth, respectively (18). Although these are promising results, the vulnerability of peptide antagonists to proteolytic attack, size restrictions limiting their tissue penetration, and the difficulty in chemically synthesizing large amounts of ΔH2 derivatives remains challenge. Our recent discovery of a novel RXFP1 ligand that is structurally distinct from relaxin-like peptides may provide new opportunities for developing RXFP1 antagonists. We identified C1q/tumor necrosis factor-related protein (CTRP) family member CTRP8 as a novel RXFP1 ligand. Importantly, a small competitor peptide derived from the closely related C1q/tumor necrosis factor-related factor 6 (CTRP6) was able to disrupt the CTRP8-induced and RXFP1-dependent migration of human glioma cells (27). This suggests a novel and as yet poorly understood regulatory network in which C1q/tumor necrosis factor-related factors, depending on the presence of resident secreting cells, can modulate RXFP1 functions in a tissue-dependent and tumor-specific manner.

## Tissue Distribution and Structure of CTRP Family Members

The family of complement C1q/tumor necrosis factor-related proteins is composed of adiponectin and 16 CTRP members (CTRP1-9, 9B, 10−15). All CTRPs are secreted proteins that get assembled into trimers and higher-order oligomers. In co-expression systems, CTRPs can also form heteromeric complexes (28–30). The C-terminal globular domain of CTRPs shares close similarity with the 3D structure of the complement component C1q and the tumor necrosis factor (TNF) homology domain present in members of the TNF family (31–33). Unlike adiponectin, which is produced at high level and almost exclusively by adipocytes, many CTRP members have broad expression profiles. CTRP members were shown to be expressed in various tissues and cell types (28, 34–45). Of particular interest for this review is CTRP8 detected by PCR in the testis and lung (29).

**Figure 1:**
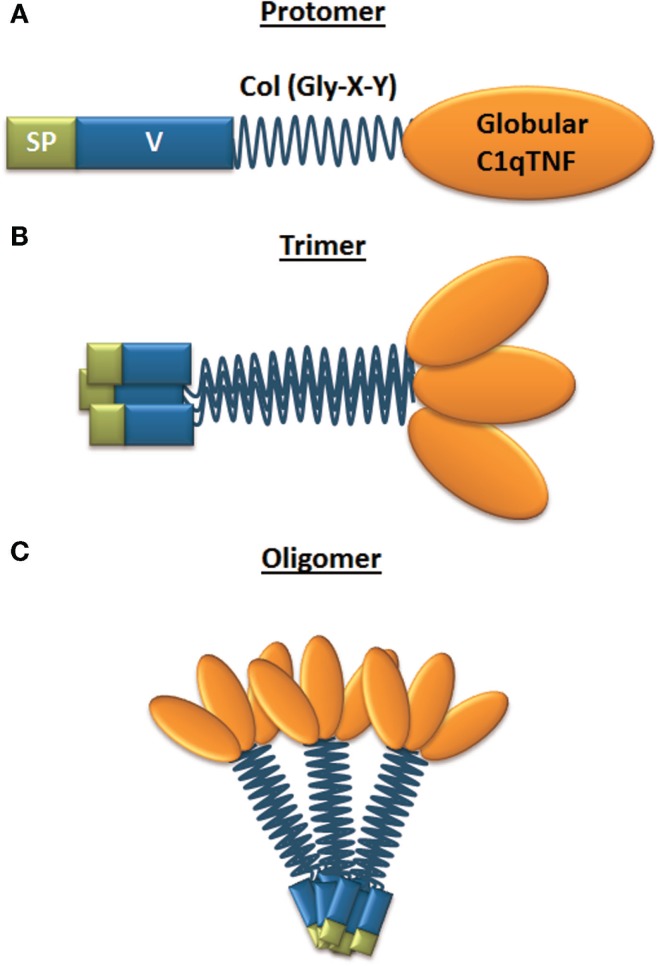
**Structure of CTRP family members**. **(A)** Domain structure of a single CTRP protomer, which is composed of a signal peptide (SP), variable region (V), collagen domain (Col – Gly-X-Y), and globular C1q/TNF domain; **(B)** CTRP are secreted and can form homotrimers; **(C)** trimers can form higher-order multimeric 3D structures composed of multiple trimers.

The structure of CTRPs is highly conserved during vertebrate evolution as determined by sequence comparison of orthologous CTRPs from zebrafish, frog, mouse, and human genomes (46). All CTRPs share a common protein structure with adiponectin, with CTRP9 showing the highest homology (54%) in the globular C1q domain to adiponectin (30). *Ctrp8* and *Ctrp9B* are both pseudogenes in the mouse genome (29). The structure of CTRPs consists of four distinct domains (Figure 1A). The *N-terminal signal peptide* facilitates protein secretion and is followed by a *short variable region*. The variable region contains one or more conserved cysteine (Cys) residues, which create disulfide bonds to facilitate higher-order multimeric protein assembly and/or secretion (28, 30, 46–49). Next, a *collagen-like domain* contains a variable number of Gly-X–Y (where X and Y indicate any amino acid; often Y is a proline or hydroxyproline) repeats, which are essential for the formation of a left-handed coiled coil structure composed of three collagen-like chains. This collagen triple helix acts as a stabilizing stalk for the formation of CTRP trimers (Figure 1B) (32). Located at the C-terminus of each collagen-like domain is a *jelly-roll* β*-sandwich folded globular domain* with 3D structural homology to complement component C1q and the tumor necrosis factor ligand family, hence the name CTRP (28, 45, 46, 50). Connected by the collagenous stalk, three such C1q-like protomers form the globular head typical for CTRP members. The collagen domain not only facilitates trimer formation but also contributes to multimerization of CTRPs into bouquet-like quaternary structures (Figure 1C) (32, 51). CTRP4 is unique among the secreted members of the C1qTNF family as it lacks the collagen domain but, instead, contains two tandem globular C1q domains connected by a short non-conserved 18 amino acid linker. CTRP4 protein is encoded by a single exon and its globular C1q domain shows highest homology (44%) to the CTRP member C1qDC1/Caprin-2 (52). The crystal structure of CTRP5 identified residues Y_152_, F_230_, and V_232_ within the globular domain as important contributors to the trimer formation and these residues are highly conserved among C1q family proteins (29, 33, 51). Exceptions are CTRP1^Y230^ and CTRP6^H230^ and CTRP8^H230^ where the phenylalanine residue F_230_ of CTRP5 is replaced by less hydrophobic His (H) or Tyr (Y) residues, suggesting potential differences in trimerization and complex stability (51). This finding is intriguing for three reasons: (i) phylogenetic analysis of currently known C1q globular domains of C1q-like family members identified a close relationship of CTRP members 1, 6, and 8 (33, 53); (ii) CTRP1 and CTRP8 share an identical peptide sequence identified as a binding motif for the relaxin receptor RXFP1 (54, 55), and (iii) we described CTRP8 as a novel ligand of RXFP1 (27).

CTRPs are subjected to posttranslational modifications. This includes *N*-linked glycan modifications for CTRP1, CTRP2, CTRP6, CTRP12, and CTRP15, whereas CTRP3, CTRP5, CTRP9, CTRP10, CTRP11, and CTRP13 contain other carbohydrate moieties (28, 30, 46–48, 50, 53, 56, 57). However, N-glycanase-sensitive glycosylation was not detected for CTRP8 (29). Also, bacterially produced (non-glycosylated) recombinant proteins, CTRP1, CTRP6, and CTRP8, retain bioactivity, suggesting that posttranslational glycan modifications are not required for some of their biological effects. Adiponectin, CTRP2, CTRP3, CTRP4, CTRP7, CTRP9, CTRP10 contain Ca^2+^ binding sites, whereas CTRP1, CTRP5, CTRP6, and CTRP8 lack a Ca^2+^-binding element (32, 49, 51). Ca^2+^ ions were reported to promote stable trimer formation and oligomerization of adiponectin (58, 59).

## CTRP Members in Cancer

Research on the role of C1q-TNF-related proteins in cancer is an emerging field and so far CTRP3, CTRP4, and CTRP6 have been associated with tumor-promoting effects. Secreted *CTRP3/cartducin* plays a role in cartilage development. Elevated protein expression of CTRP3/cartducin in mouse osteosarcoma cell lines was shown to promote cell proliferation in a dose-dependent manner. The MAPK/ERK kinase 1/2 (MEK1/2) inhibitor, U0126, prevented the mitogenic effect indicating that CTRP3 induces cell proliferation via ERK1/2-signaling (60). CTRP3 was also shown to induce migration of mouse endothelial cells in an ERK1/2-dependent manner (39) suggesting a role in angiogenesis. The receptor mediating the effects of CTRP3/cartducin is unknown. HeLa and HEK293 cells were used to show that *CTRP4* functions as tumor-promoting inflammatory regulator. CTRP4 overexpression increased NFκB activation in a dose-dependent manner and induced transcriptional activity of the NFκB target TNF-α. In human HepG2 hepatocarcinoma cells, secreted CTRP4 and recombinant CTRP4 caused enhanced STAT3^Tyr705^ phosphorylation and increased IL6 and TNF-α secretion dose-dependently with a maximal stimulation at 4 ng/ml. Interestingly, increased expression of CTRP4 upon IL-6 exposure indicated a positive feedback regulation in cancer cells (61). Immunoreactive *CTRP6* was detected in human hepatocellular carcinoma tissue specimens and was localized to hepatocellular carcinoma cells and to endothelial cells within the tumor. Recombinant CTRP6 increased Akt phosphorylation in isolated liver endothelial cells and this signaling was mediated via the C-terminal C1q domain of CTRP6. Indeed, HepG2 xenografts with exogenous expression of CTRP6 showed increased tumor angiogenesis and reduced necrosis (62).

## CTRP8 is a Novel RXFP1 Ligand in Glioblastoma

CTRP8 is evolutionarily highly conserved and secreted as a homotrimer or heterotrimer with the C1qTNF family member C1q-related factor (CRF) (63); the latter also forms heterotrimers with CTRP1, CTRP9, and CTRP10 when co-expressed in cells (29). Until recently, CTRP8 was the least understood C1q-TNF-related protein member, in part, because Ctrp8 is a pseudogene in mice and PCR analysis revealed restricted expression of CTRP8 in human lung and testis (29). We recently identified CTRP8 as a novel ligand for RXFP1 in human glioblastoma cells (27). Human patient-derived glioblastoma (GB) cells and established GB cell lines express RXFP1, but lack the classical RXFP1 ligands, RLN1 and RLN2. We demonstrated the expression and secretion of CTRP8 in patient GB cells and discovered that RXFP1 serves as a novel receptor for CTRP8 in GB. CTRP8 and RLN2, as well as two biologically active peptides homologous to a peptide sequence within the N-terminal region of the C1q globular domain of human CTRP8, P59, and P74 (54, 55), activated RXFP1 by inducing cAMP signaling and PI3K–PKCζ/PKCδ–ERK1/2 signaling in GB cells. The RXFP1-negative U251 GB cell line and HEK293 cells devoid of RXFP1 with exogenous expression of the related receptor RXFP2 did not respond with increased cAMP levels demonstrating a specific RXFP1-mediated signaling. Furthermore, the increased cell motility by CTRP8, P59, and P74 showed a dose–response and was critically dependent on RXFP1. RXFP1 activation by CTRP8, P59, and P74 increased cathepsin-B protein production and secretion, which mediated the RXFP1-induced enhanced GB cell invasiveness through laminin matrices. Specific inhibitors for PKCζ, PKCδ, PI3K, and cathepsin-B and RNAi-mediated RXFP1 knockdown abolished GB invasiveness. We demonstrated the interaction between RXFP1 and CTRP8 by co-immunoprecipitation of epitope-tagged HA-RXFP1 and CTRP8-His in HEK293 cells. Our structural simulation studies predicted that the amino acid sequence “YAAFSVG” present in the P59 and P74 peptides and located within the N-terminal C1q globular domain of CTRP8 were likely interacting with the leucine-rich repeats (LRR) 7 and 8 of RXFP1 (27). We dismissed the possibility of the formation of CTRP8/CRF heterotrimers because GB cells were devoid of the CTRP8 hetero-oligomerization partner CRF (29). Importantly, competitive binding assays demonstrated that a small peptide derived from the N-terminal region of the globular C1q domain of human CTRP6 successfully blocked the PI3K–PKCζ/PKCδ-mediated increase in cathepsin B secretion and cell motility (27).

## Summary and Prospective Goals

The discovery of CTRP8 as RXFP1 agonist in brain cancer is novel for a number of reasons: (i) RXFP1 is the first receptor to be identified for any of the CTRP members; (ii) CTRP8 is the first RXFP1 ligand, which is not structurally related to the relaxin-like family; (iii) the CTRP8–RXFP1 ligand–receptor system is a novel player in brain tumor; (iv) our discovery of a competitor peptide resembling a linear peptide sequence at the transition from the collagen- to the C1q globular domain of CTRP6, a close relative of CTRP8, provides first intriguing evidence for a regulatory network of CTRP factors modulating RXFP1 functions in a tissue-specific context. These findings are the exciting start of an emerging field in CTRP and RXFP1 research with the potential to link metabolic and immunological functions of CTRP members with molecular mechanisms in cancer (45). Future cancer research activities will elucidate the molecular signaling mechanisms and functional relevance of CTRP-derived RXFP1 regulation in a variety of tumors. The use of CTRP-based peptides capable of blocking CTRP8-mediated actions is currently tested for potential clinical applications.

## Conflict of Interest Statement

The authors declare that the research was conducted in the absence of any commercial or financial relationships that could be construed as a potential conflict of interest.
